# Novel Palmitoyl Pentapeptides: Self-Assembly, Collagen Stimulation, Antioxidant Activity, and Influence of Skin Microbiota Health

**DOI:** 10.1021/acsomega.5c12526

**Published:** 2026-03-20

**Authors:** Juliane N. B. D. Pelin, Lucas R. de Mello, Raquel Allen Garcia Barbeto Siqueira, Matheus de Souza Alves, Valeria Castelletto, João Francisco Almeida, Jani Seitsonen, Patricia Santos Lopes, Vânia Leite-Silva, Newton Andreo-Filho, Ian W. Hamley

**Affiliations:** a Departamento de Ciências Farmacêuticas, 28105 Universidade Federal de São Paulo, Diadema, São Paulo 09913-030, Brazil; b Department of Chemistry, 6816 University of Reading, Reading RG6 6AD, United Kingdom; c Programa de Pós-Graduação em Medicina Translacional, Departamento de Medicina, Escola Paulista de Medicina, 28105 Universidade Federal de São Paulo, São Paulo 04021-001, Brazil; d Centro de Ciências Naturais e Humanas, Universidade Federal do ABC, Santo André 09210-580, Brazil; e Nanomicroscopy Center, 174277 Aalto University, Puumiehenkuja 2, Espoo FIN-02150, Finland

## Abstract

New lipopeptide analogues of C_16_–KTTKS, containing tyrosine (C_16_–KTTKY) and glutamic acid (C_16_–KTTKE) residues, were characterized by physicochemical and biological assays to understand their collagen stimulation and ability to control skin commensal microorganism growth. The presence of nanotapes based on stacked lipopeptide lamellae was confirmed by cryogenic transmission electron microscopy and small-angle X-ray scattering. Variations in zeta potential as a function of lipopeptide concentration indicated the electrostatic stability of C_16_–KTTKE, while C_16_–KTTKY was stable at a lower concentration, with a similar aggregation state. Circular dichroism spectra revealed a transition from random coil to β-sheet for both peptides with increasing temperature (up to 50 °C). Significant statistical reductions in cell viability below 70% were observed at concentrations above 0.00625 wt % for C_16_–KTTKE and 0.00156 wt % for C_16_–KTTKY, respectively. At higher lipopeptide concentrations, C_16_–KTTKE promotes a decrease in total collagen production by human dermal fibroblasts; however, it has antioxidant properties. In contrast, C_16_–KTTKY stimulates a considerable increase in the level of total collagen production. The lipopeptides were found to stimulate *S. epidermidis* growth, a microorganism very important for skin microbiota health. Therefore, both lipopeptides have interesting characteristics as active ingredients in antiaging cosmetic products.

## Graphical abstract

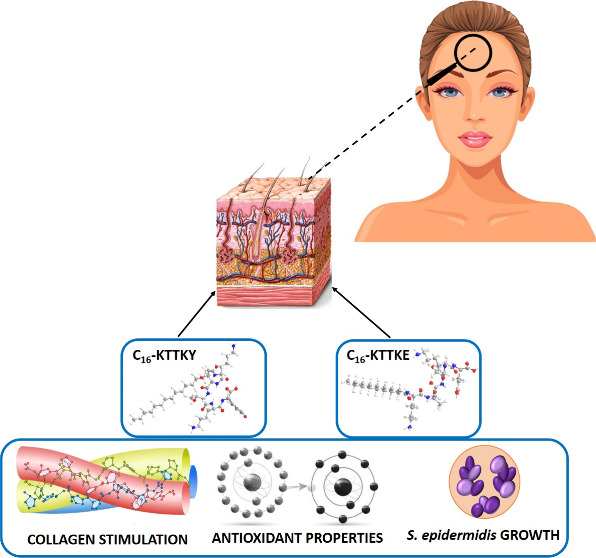

## Introduction

Each year, new functionalities are discovered for lipopeptides, covering several areas of application, including medicine,^1–3^ pharmaceuticals,^4,5^ cosmetics,^5–7^ chemistry,^1,8,9^ and engineering.^10–13^ The interest in these systems is correlated to the simplified mimicry of compounds present *in vivo* and in the process of self-organization. Lipopeptides that stimulate the proliferation of collagen production are gaining prominence in tissue engineering, including applications including skin repair after trauma, such as burns and surgery; and in cosmetology, in order to accelerate the process of cell renewal, giving better structural characteristics to mature skin, reducing the signs of aging, such as wrinkles.^5,14–17^

Collagen production is important because this protein is correlated with skin integrity. During the skin aging process, there is a large decrease in elastin and collagen and a reduction of fibroblast numbers in the dermis, resulting in fine lines and wrinkles.^18–21^ Skin aging is a multifactorial process, involving pollution, sedentary lifestyle, excessive consumption of alcohol, and photoaging from exposure to UV light, high ingestion of sugar, or harmful substances. These processes promote the enhancement of ROS (reactive oxygen species), a major factor in the disruption of cellular integrity.^22–26^

It is a significant challenge to recover skin that has already undergone these degradation processes, without using invasive procedures that promote significant changes in facial features. Use of compounds with the ability to repair skin via stimulation of the production of collagen in the dermis is attractive for such uses, especially for topical application. An advantage of working with lipopeptides as an active ingredient in antiaging cosmetic formulations is that they present a lower risk of developing allergic reactions to the skin.^27^ Peptides developed for antiaging skincare treatments may be lipidated, and this introduces amphiphilicity, which may lead to self-assembly propensity. In turn, self-assembly may influence bioactivity and effective permeation through the *stratum corneum*. To enable transport of these lipopeptides, avoiding cytotoxic processes, formulations that encapsulate active ingredients within lipid NPs have been developed as delivery systems.^28,29^

Cosmetics containing solid lipid nanoparticles (SLN) and nanostructured lipid carriers (NLC) can aid the permeation of active/bioactive ingredients.^28–31^ However, the optimization of these systems for the treatment of skin disorders is still a challenging process since fine control of their physicochemical properties must be achieved to enhance their nanocarrier action. A major advantage of these systems includes the formation of an occlusive layer on the surface, providing greater hydration. In addition, they provide a more prolonged release of active/bioactive ingredients through a more stable and controlled process, enabling penetration into deeper layers of the skin. They also allow greater selectivity and cutaneous localization of the selected active ingredients, through biocompatible routes and with low irritation potential.^30,32^

Lipopeptide C_16_–KTTKS (C_16_: palmitoyl or hexadecyl, KTTKS: lysine–threonine–threonine–lysine–serine) is a so-called matrikine, an extracellular matrix collagen pro-protein fragment, capable of stimulating type I, II, and III collagen and fibronectin production in the skin inhibition.^33–37^ There are many synthetic peptides that can improve the skin integrity;^37,38^ however, KTTKS analogues are the focus of ongoing research, including palmitoyl (C_16_) variations of KTTKS peptides (Pal-KTTKS–OH, Pal-KTTKS-NH_2_, Pal-KTTRS–OH, Pal-KTTRS-NH_2_, Pal-RTTRS–OH, Pal-RTTRS-NH_2_, Pal-RTTKS–OH, Pal-RTTKS-NH_2_), which demonstrated good activity as plasmin inhibitors, with no cytotoxicity with fibroblasts.^39^ These properties were enhanced for lipidated peptides, which promoted an increase in thermal stability and promoted polyproline II-like triple helical structure formation, which mimics the collagen nanostructures.^40^

C_16_–KTTKS helps to inhibit kinases such as elastase, collagenase, and hyaluronidase enzymes and minimize damage to essential biomolecules by antioxidant mechanisms.^41^ In this way, the C_16_–KTTKY and C_16_–KTTKE structures (Figure 1) were strategically designed, considering tyrosine (Y) and glutamic acid (E) as the terminal amino acids. Aiming to develop multifunctional molecules, inclusion of a tyrosine residue can potentially impart antimelanogenic properties, reducing melanin release and promoting a lightening effect on dark skin spots. Also, tyrosine has antioxidant properties and can be correlated with the production of hormones from the thyroid, adrenal, and pituitary glands, playing an important role in metabolism.^42–44^ Alternatively, incorporation of glutamic acid in the peptide sequence may help to inhibit inflammatory cytokines, reducing skin damage.^45^ It is important to mention that glutamic acid can be converted to glutamine by glutamine synthetase, and this natural mechanism provides a molecule associated with energy production and redox homeostasis, protecting the body against aging injuries.^46^

**Figure 1:**
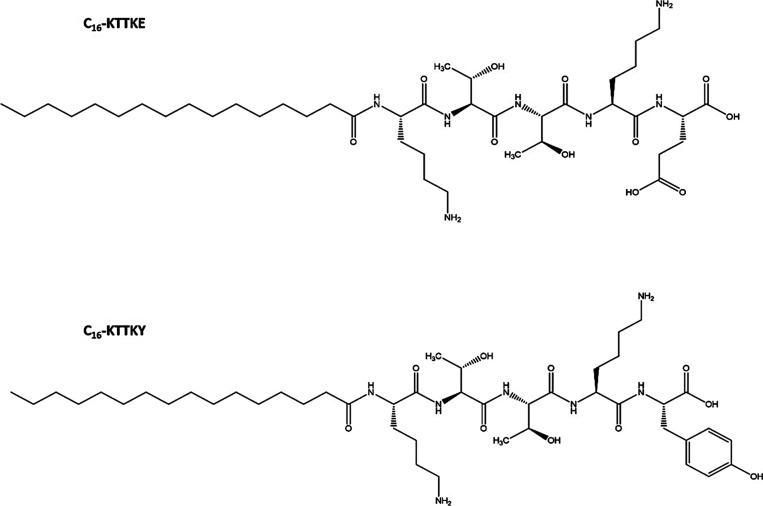
Molecular structures of C_16_–KTTKY and C_16_–KTTKE lipopeptides.

Here, we compare the self-assembly, conformational properties, and biological behavior of the lipopeptides shown in Figure 1 with C_16_–KTTKS. To the best of our knowledge, these peptides are novel molecules, which bring additional characteristics due to the presence of tyrosine and glutamic acid residues, with the aim of creating multifunctional products for cosmetics and tissue engineering.

## Materials and Methods

### Materials

The lipopeptides were synthesized by Aminotech Pesquisa e Desenvolvimento, Brazil, with purity evaluated by high-performance liquid chromatography (HPLC) > 95.0%. The molecular weight of each lipopeptide was determined by mass spectrometry: C_16_–KTTKY (Palmitoyl-Lys-Thr-Thr-Lys-Tyr) 878.6 g mol^–1^ (expected: 878.2 g mol^–1^) and C_16_–KTTKE (Palmitoyl-Lys-Thr-Thr-Lys-Glu) 843.6 g mol^–1^ (expected: 844.1 g mol^–1^). All lipopeptide solutions were prepared with water purified using a Millipore Direct-Q System with a resistivity of 18.2 mΩ cm^–1^ (at 25 °C) and a TOC below 10 ppb.

### Fluorescence Spectroscopy

Values of lipopeptide critical aggregation concentration (*cac*) were determined by fluorescence spectra, recorded with a Varian Cary Eclipse fluorescence spectrometer with samples in 4 mm inner quartz cuvettes. Pyrene assays were performed using 2.5 × 10^–4^ to 0.1 wt % lipopeptide, in 2.2 × 10^–5^ wt % pyrene solution. The samples were excited at λ_ex_ = 339 nm, and the fluorescence emission was measured for λ = 360–600 nm, at room temperature.

### Circular Dichroism (CD)

CD spectra were obtained using a Jasco-815 CD spectropolarimeter (Jasco Co). Solutions containing 0.5 wt % of lipopeptide were scanned at 20 to 50 °C in a quartz cuvette with a thickness of 1.0 mm. The spectra were recorded with absorbance *A* < 2 at any measured point, scanning at 50 nm min^–1^. A water background CD signal was used to subtract the baseline from the experimental data.

### Dynamic Light Scattering

Size and zeta potential measurements were performed using a Zetasizer Nano ZS instrument. For zeta potential measurements, 0.5 wt % lipopeptide water solutions were used, and size assays were made considering 0.05, 0.08, and 0.5 wt % solutions.

### Cryogenic Transmission Electron Microscopy (Cryo-TEM)

Imaging was carried out using a field emission cryoelectron microscope (JEOL JEM-3200FSC), operating at 200 kV. Images were taken in bright field mode and using zero-loss energy filtering (omega type) with a slit width of 20 eV. Micrographs were recorded using a Gatan Ultrascan 4000 CCD camera. The specimen temperature was maintained at −187 °C during the imaging. Vitrified specimens were prepared using an automated FEI Vitrobot device using Quantifoil 3.5/1 holey carbon copper grids with a hole size of 3.5 μm. Just prior to use, grids were plasma cleaned using a Gatan Solarus 9500 plasma cleaner and then transferred into the environmental chamber of a FEI Vitrobot at room temperature and 100% humidity. Thereafter, 3 μL of sample solution was applied on the grid, and it was blotted twice for 5 s and then vitrified in a 1/1 mixture of liquid ethane and propane at a temperature of −180 °C. The grids with vitrified sample solution were maintained at liquid nitrogen temperature and then cryo-transferred to the microscope.

### Small-Angle X-ray Scattering (SAXS)

SAXS experiments were performed on beamline B21 ^47^ at Diamond Light Source (Harwell, UK). The sample solutions were loaded into the 96-well plate of an EMBL BioSAXS robot and then injected via an automated sample exchanger into a quartz capillary (1.8 mm internal diameter) in the X-ray beam. The quartz capillary was enclosed in a vacuum chamber to avoid parasitic scattering. After the sample was injected into the capillary and reached the X-ray beam, the flow was stopped during the SAXS data acquisition. Beamline B21 operated with a fixed camera length (3.9 m) and fixed energy (12.4 keV). The images were captured using a PILATUS 2 M detector. Data processing was performed using dedicated beamline software ScÅtter. Data are presented as a function of *q* = 4πsinθ/λ, where 2θ is the scattering angle.

### Cell and Tissue Culture

Human Dermal Fibroblast cells from adult donors (HDFa) (Sigma-Aldrich – UK) were cultivated in DMEM/F12 (Dulbecco’s modified Eagle medium F12), with the addition of 5% fetal bovine serum (FBS), 20 mM HEPES, GlutaMAX (Gibco – UK), and antibiotic–antimycotic (Gibco – UK). The cells were maintained inside a cell incubator with the temperature set at 37 °C, and under an atmosphere of 5% carbon dioxide (CO_2_).

### MTT Assays

Cytotoxicity of lipopeptides was determined using 3-(4,5-dimethylthiazol-2-yl)-2,5-diphenyltetrazolium bromide (MTT) assays (Merck Sigma-Aldrich, UK). Initially, human dermal fibroblasts (HDFa) cells were maintained in supplemented DMEM/F12, and after reaching confluence, the cells were harvested and seeded at a confluence of 5 × 10^3^ cells into 96-well plates. The plates containing HDFa cells were incubated for 72 h in DMEM/F12 with different concentrations of lipopeptide (0.0002–0.05 wt %). After the 72 h incubation, the plates were washed 3 times with PBS (phosphate-buffered saline), and 100 μL of media without serum +0.05 wt % MTT was added to the cells. The plates were again incubated for 4 h, protected from the light at 37 C. The media was removed, and the resulting formazan crystals were dissolved using 100 μL of DMSO (dimethyl sulfoxide) for 30 min at 37 °C, protected from the light. The sample absorbance was read at 570 nm using an Infinite 50 Tecan instrument (Tecan, UK).

### Picrosirius Red Assay for Collagen Detection

To determine the production of collagen, 5 × 10^3^ HDFa cells were seeded into 96-well plates, using DMEM/F12 media and left to attach overnight inside a cell incubator with a 5% CO_2_ atmosphere at 37 °C. The next day, samples consisting of different concentrations of the evaluated lipopeptides dissolved in media were added to the plates and incubated for 72 h inside the cell incubator. The controls for this assay were incubated only in the media. After the 72 h incubations, the cells were washed 3 times with PBS and fixed with ice-cold ethanol for 20 min inside an ultrafreezer for collagen fixation. A final incubation with Direct red 80 (Alfa Aesar – UK) diluted in picric acid 1% at a concentration of 0.1 wt % was conducted at 4 °C overnight, for collagen staining. The plate was washed with ultrapure water to remove any excess dye, and 100 μL of 1 M sodium hydroxide (NaOH) was added to each well to dilute the stained collagen fibers. The resulting absorbance was measured at a fixed wavelength of 492 nm, and the total collagen deposited was calculated using a calibration curve made with known standards of rat tail collagen (Sigma-Aldrich – UK).

### Stimulation of Type I Collagen Production

Human fibroblast cell cultures were used, maintained in culture with DMEM supplemented with additives, in an incubator at 37 °C and 5% CO_2_, and handled inside a laminar flow hood.^48^ The cells were distributed in a culture plate, and 1 wt % aliquots of the lipopeptides were used in the culture medium. The control group was supplemented with culture medium. The solutions containing the control and sample groups were applied to the cell culture, 100 μL per well in triplicate for each group, followed by incubation in an incubator at 37 °C and 5% CO_2_ for 48 h. After this time, the amount of collagen produced by the fibroblasts was evaluated using a specific marker as described in the literature.^48^ The sample absorbance was read at a wavelength of 540 nm. The results were evaluated by using Graphpad Prism 5 software. The result of the control group was normalized to 100%, and each sample was compared to it. Statistical analysis for comparison between groups was performed using a one-way ANOVA test and a Bonferroni post hoc test, with a statistical significance level considered less than 0.05.

### Growth Curve of Microorganisms

The strain used was *Staphylococcus epidermidis* (ATCC 12228*).* The growth curve of *S. epidermidis* was obtained using Brain–Heart Infusion Broth (BHI) (Lab. Companion, Naperville, IL, USA) at 37.5 °C and 150 rpm in a shaker inside an incubator (Ethik Technology, São Paulo, Brazil). Briefly, to obtain the growth curve of microorganisms, the determination of the number of microorganisms in the suspension was achieved by measuring the absorbance at a wavelength of 650 nm with a spectrophotometer (Eppendorf AG, Hamburg, Germany), and the populations were measured every 24, 48, and 96 h.

### Inhibition Test in Agar

To perform the agar diffusion test using the well method, 20 mL of antibiotic medium was added to Petri dishes for the cultivation of bacteria. The inoculum volumes determined to reach 10^6^ CFU/mL bacteria were spread evenly with a sterile swab over the entire surface of the agar. Filtered 20 μL aliquots of the lipopeptide solutions were used. After forming 7 mm diameter wells, 20 μL of the samples was added to the wells to diffuse the substances into the agar. The plates with bacteria were incubated at 37 °C for 24 h. After the incubation period, 5 mL of nutrient agar with 0.1% of the dye 2,3,5-triphenyltetrazolium chloride (TTC) (Sigma-Aldrich, Merck, Darmstadt, Germany) was added to stain the bacterial cells. After 48 h of incubation, the responses to the samples were evaluated by measuring the microorganism growth. Paper disks of chloramphenicol were used as a positive control (Sensidisc DME, São Paulo, Brazil).

### Microdilution Test

For the broth microdilution test, standardized concentrations of peptides were prepared, following the recommendations of the CLSI M07 and ISO 20776–1 standards. From the serial dilutions, the concentrations tested in the wells were 3.0, 1.5, 0.75, 0.375, 0.187, 0.093, 0.046, and 0.022 wt % of lipopeptide, measurements being performed in quadruplicate on the plate. All steps were carried out in a laminar flow cabinet. Each lipopeptide was tested against 10^6^ CFU/mL of *S. epidermidis*. The plates tested were incubated at 37.5 °C for a maximum of 24 h. After the incubation period, optical densities were read using a microplate reader (BioTek, model: Synergy HT, software: Gen5, Agilent, Santa Clara, CA, USA) at a wavelength of 600 nm.

### Antioxidant Activity Assay in Monolayer

HaCaT cells (5 × 10^4^ cells/well) were seeded in black 96-well plates with transparent bottoms (0.28 cm^2^/well) and incubated for 48 h at 37 °C, to allow for cell adhesion and growth. Samples were diluted in PBS and tested at two concentrations previously determined (data not shown) to assess safety and potential cytotoxic effects under extreme conditions. After 48 h, the culture medium was removed, and the cells were washed with 100 μL of PBS. The fluorescent probe DCFH-DA (2’,7’-dichlorofluorescein diacetate, 2000 μM; 50 μL/well) was then added, followed by incubation for 30 min at 37 °C. The cell control (CC) received only PBS, representing the basal condition. Hydrogen peroxide (H_2_O_2_, 4 M) was added as an oxidative stress inducer, except for the CC and DCFH controls, which received PBS only. The samples (C_16_–KTTKE and C_16_–KTTKY) and the positive control (resveratrol, 250 μM) were then added to the respective wells. The plates were covered with aluminum foil and incubated for an additional 30 min at 37 °C. After this period, the contents were aspirated and replaced with 100 μL of PBS, followed by shaking for 10 min to ensure homogenization. Fluorescence was measured using a microplate reader (BioTek Synergy HT Multimode, Gen5 software) with an excitation at 485 nm and emission at 538 nm. The fluorescence intensity of DCF was used as an indicator of intracellular ROS production, allowing the assessment of the antioxidant activity of the tested samples.

## Results

The critical aggregation concentration (*cac*) values of the lipopeptides were obtained by fluorescence assays of lipopeptide aqueous solutions, using pyrene as a probe that is well-known to interact with hydrophobic regions of self-assembled nanostructures, intensifying its fluorescence signal.^49,50^ Figure 2 shows the original fluorescence emission spectra and the fluorescence intensity as a function of the peptide concentrations, which revealed *cac* values (8.6 ± 0.5) × 10^–3^ wt % and (7.3 ± 0.5) × 10^–3^ wt %, respectively, for C_16_–KTTKE and C_16_–KTTKY. These results show a slight increase in hydrophilicity of lipopeptides when compared with C_16_–KTTKS (*cac* = 5.6 × 10^–3^ wt %).^35^

**Figure 2:**
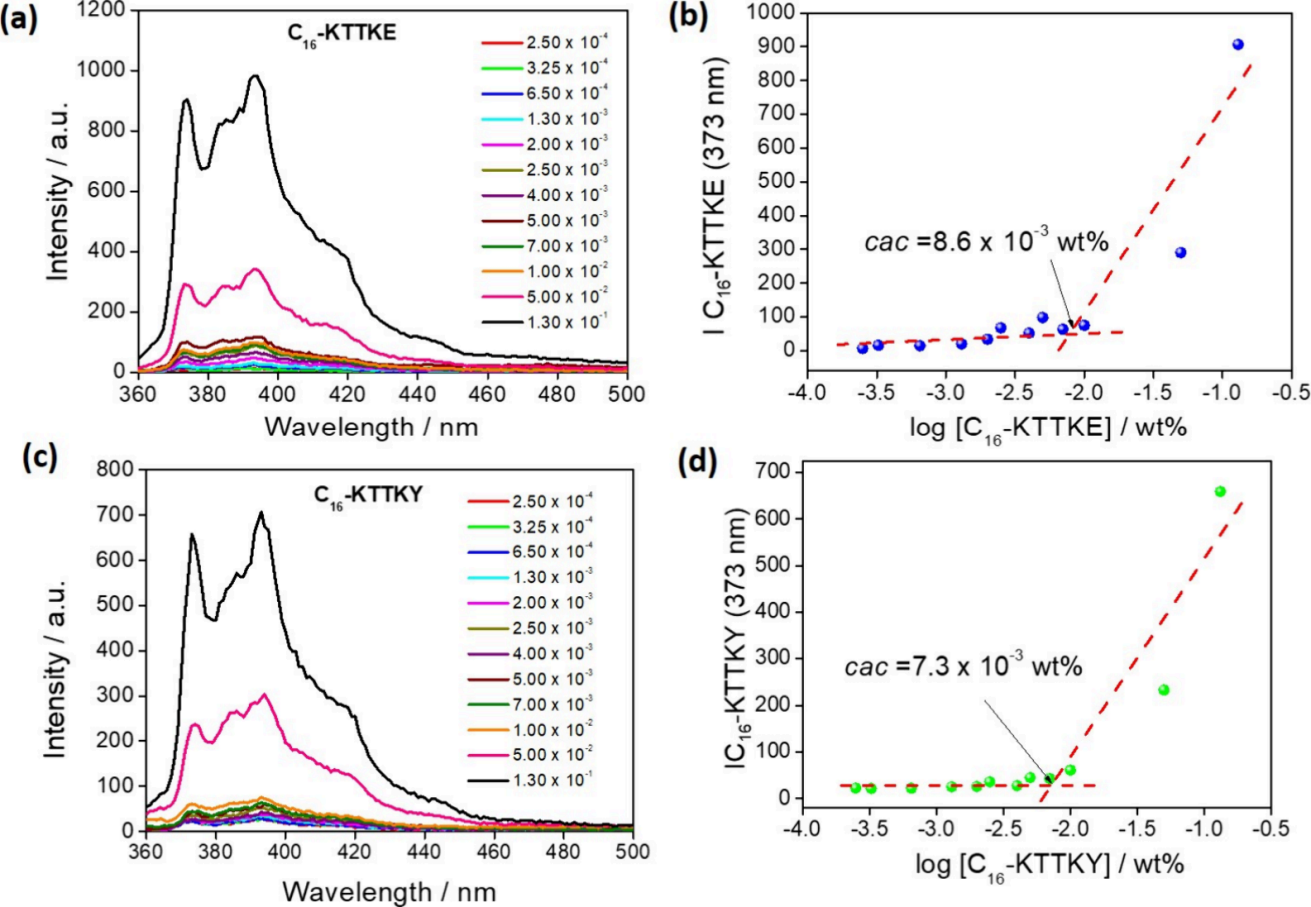
Emission spectra for solutions containing 2.2 × 10^–5^ wt % pyrene and solutions with different concentrations (2.5 × 10^–4^ to 0.1 wt %) of (a) C_16_–KTTKE and (c) C_16_–KTTKY with λ_excitation_ = 339 nm and slits of 2.5/5. Intensity of pyrene fluorescence as a function of concentration for (b) C_16_–KTTKE and (d) C_16_–KTTKY.

To probe secondary structure, circular dichroism (CD) spectra were obtained above the determined *cac* values, and the spectra are presented in Figure 3. The spectrum of C_16_–KTTKE (Figure 3a) at 20 and 30 °C contains two negative peaks at 200 and 215 nm, the latter indicating the presence of β-sheet conformation. Increasing temperature causes a transition to a disordered conformation.^51^ From Figure 3b, the spectra at 20 to 40 °C of C_16_–KTTKY show a positive peak at ∼180 nm (α-helix structure) and negative peaks at 200–205 nm (π–π* transition) and 218 nm (n−π* transition), respectively. The strong negative peak at around 210 nm can be attributed to π–π* transition of the phenol moiety of tyrosine, overlapping with the electronic transition of the amide groups.^52^ At 50 °C, this system contains two negative peaks at 190 and ∼220 nm, indicating a β-sheet conformation.

**Figure 3:**
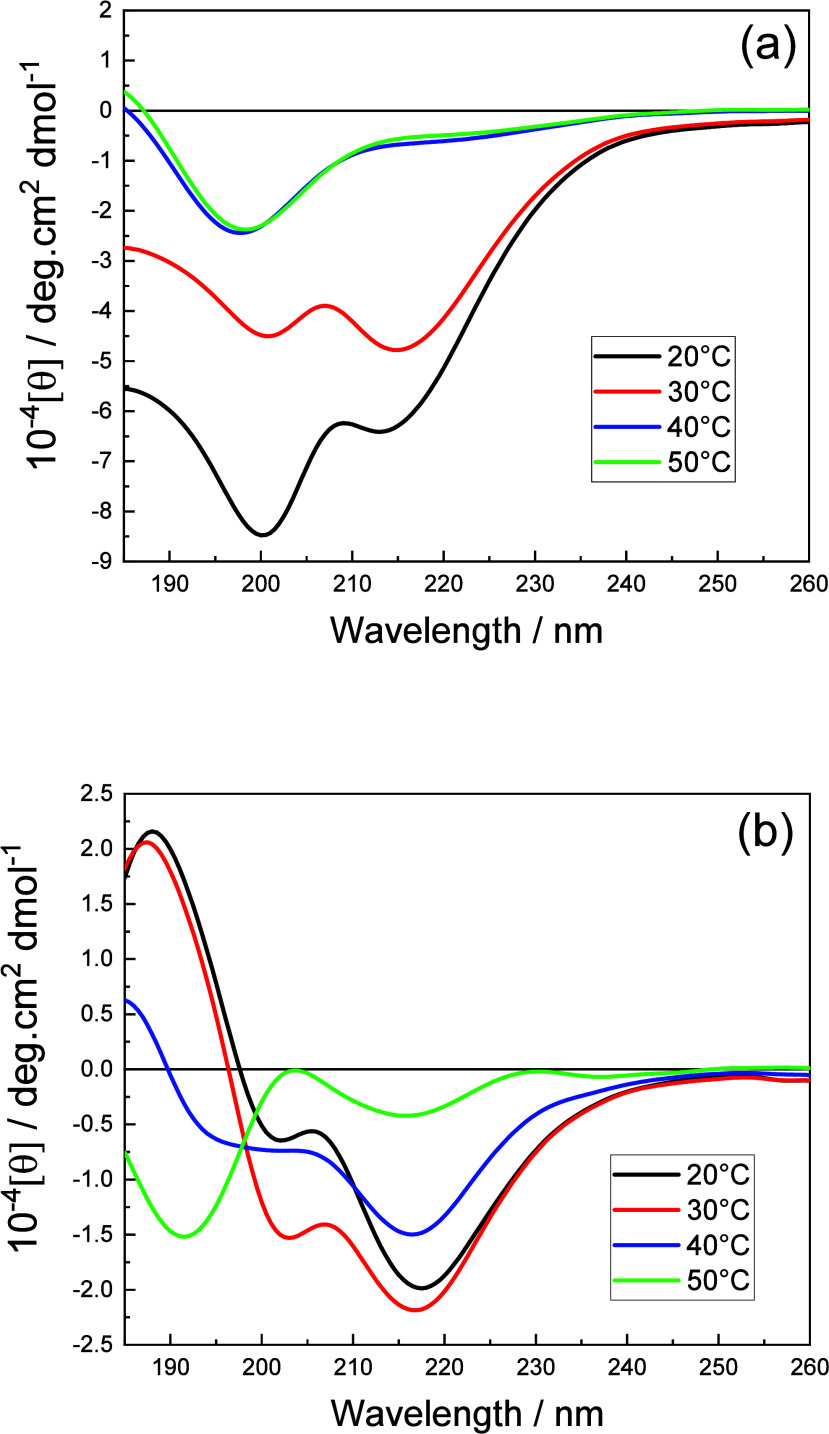
CD spectra for 0.5 wt % aqueous solutions of (a) C_16_–KTTKE and (b) C_16_–KTTKY, at the temperatures indicated.

The self-assembly of the C_16_–KTTKE and C_16_–KTTKY systems was examined using a combination of scattering and microscopy techniques, for solutions above the *cac*. The zeta potential was measured and found to be highly dependent on lipopeptide concentrations, as evident from the data in Table 1. The systems were stable at 0.05 wt % concentration, due to their zeta potential values above 30 mV,^53^ and they are positively charged. However, with increasing concentration, a gradual decrease in surface charge was observed, especially for C_16_–KTTKE at 0.5 wt % and pH ≈ 7, attributed to deprotonated carboxyl groups, which also showed high stability.

**Table 1:** Zeta Potential Results for 0.05, 0.08, and 0.5 wt % Aqueous Solutions of C_16_–KTTKE and C_16_–KTTKY, at 25 °C

C_16_–KTTKE		
0.05 wt %	0.08 wt %	0.5 wt %
+51.8 mV	+31.4 mV	–25.6 mV

C_16_–KTTKY		
0.05 wt %	0.08 wt %	0.5 wt %
+60.1 mV	–3.9 mV	–1.3 mV

A significant reduction in zeta potential was observed for C_16_–KTTKY at higher concentrations, indicating that structural modifications may have favored the exposure of the phenolic group present in the tyrosine residue. Despite being negative, the zeta potential values are close to neutral at pH ≈ 7, exhibiting behavior similar to that of collagen.^54^

The self-assembled nanostructures of C_16_–KTTKE and C_16_–KTTKY were examined using cryo-TEM. The images presented in Figure 4 show that both samples form extended tape-like structures, similar to C_16_–KTTKS.^40^ There are notable differences between the two samples here, though. Lipopeptide C_16_–KTTKE forms twisted tapes, whereas C_16_–KTTKY forms flat crystal-like tapes, some of which have a striped appearance. This was examined in more detail, and images along with cross sections showing the periodicity of the stripes are shown in SI Figure S1. From the cross sections as well as the FFT of selected areas, the stripe period was found to be 5.3–5.8 nm. This is close to the lamellar spacing determined from SAXS and suggests that the stripes arise from the lamellar periodicity, some tapes surprisingly showing this structure “side-on”. Stripes with a 5 nm spacing were previously reported for C_16_-ETTES,^55^ and 4.2 nm striped nanotapes were observed for C_16_–KTTKS in the presence of the surfactant sodium dodecyl sulfate.^56^

**Figure 4:**
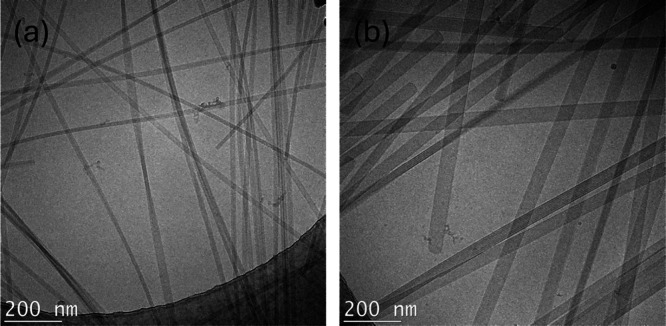
Cryo-TEM images from 1 wt % solutions of (a) C_16_–KKTKE and (b) C_16_–KTTKY.

Cryo-TEM was complemented with SAXS. The SAXS intensity profiles in Figure 5 show features of nanotapes with bilayer stacking of the lipopeptides. For C_16_–KTTKE, the data can be fitted using a model of a Gaussian bilayer^57^ (Figure 5) used previously to fit SAXS data for various nanotape-forming lipopeptides.^17,55^ Due to the broad Bragg peak at high *q*, a structure factor term was included using the Caillé model for fluctuating lamellae.^58^ The fit parameters are listed in Table S1 and indicate a tape comprising, on average, two layers with a spacing *d* = 47 Å. In contrast, the data for C_16_–KTTKY shows a series of Bragg reflections at high *q*, which are not evenly spaced and so cannot be fitted to a single lamellar structure factor. The peaks at *q* = 0.13 Å^–1^ (broad, possibly two not fully resolved peaks), *q* = 0.181 Å^–1^, *q* = 0.231 Å^–1^, and *q* = 0.271 Å^–1^ correspond to spacings *d* = 48 Å, *d* = 34.7 Å, *d* = 27.2 Å, and *d* = 23.2 Å. Since the cryo-TEM images show nanotape structures, these are assigned to coexisting lamellar structures with different degrees of hydration. For comparison, we previously reported a lamellar spacing *d* = 52.5 Å for C_16_–KTTKS^59^ and *d* = 53 Å for hydrated layers of C_16_-ETTES, with an additional *d* = 30 Å spacing assigned to dehydrated bilyers.^55^

**Figure 5:**
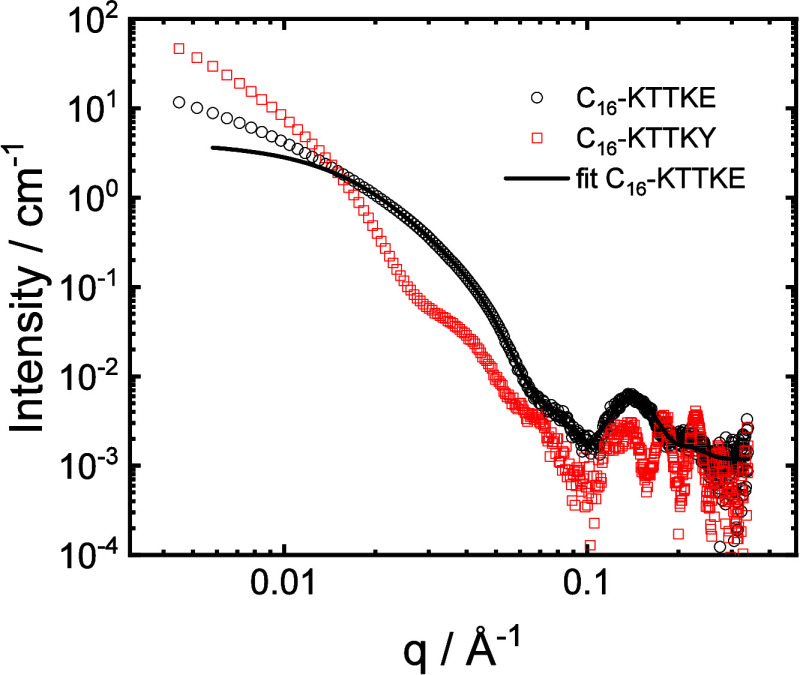
SAXS data for 1 wt % solutions of C_16_–KTTKE and C_16_–KTTKY, as indicated. The data for C_16_–KTTKE have been fitted with a bilayer form factor model, as described in the text. For ease of visualization, only every 5th data point is shown.

The cytocompatibility of each lipopeptide was determined using the MTT assay, a colorimetric assay that measures the metabolism of cells due to the capacity of living cells to reduce MTT inside the cytosol, which is an indicator of overall cell health.^60^ The MTT assay is a colorimetric method that evaluates cell viability based on the ability of metabolically active cells to reduce the tetrazolium salt MTT into insoluble formazan crystals via mitochondrial oxidoreductase activity. The amount of formazan produced is proportional to the number of viable cells and is quantified spectrophotometrically.^60^

The assay data presented in Figure 6 shows that the lipopeptide C_16_–KTTKE exhibited statistically significant cytotoxicity when compared to the control at concentrations of 0.003125 wt % or more, as indicated by the ANOVA test with Bonferroni correction for multiple comparisons (Figure 6a). For C_16_–KTTKY, a significant reduction in cytocompatibility was observed for concentrations above 0.0004 wt % (Figure 6b).

**Figure 6:**
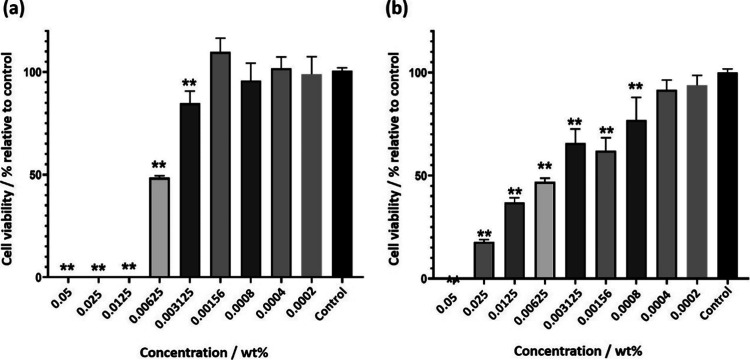
Cell viability (relative to control, media only) from MTT assays for (a) C_16_–KTTKE and (b) C_16_–KTTKY. The statistical test used was ANOVA, *n* = 3 with Bonferroni correction multiple comparisons with ** *p* < 0.01.

The results indicate that there are significant statistical reductions in cell viability below 70% at concentrations exceeding 0.00625 wt % for C_16_–KTTKE or 0.00156 wt % for C_16_–KTTKY, respectively. Comparing with C_16_–KTTKS lipopeptide, Vitali et al. recorded 78% cell viability, after 48 h treatment, using 3T3-NIH fibroblasts.^61^

The collagen production of HDFa cells exposed to the lipopeptides was quantified using the picrosirius red technique, which is a colorimetric assay used to stain collagen fibers and enables quantification by absorbance.^62^ Figures 7a,b show the total amount of collagen deposited by HDFa in the plate for C_16_–KTTKE and C_16_–KTTKY, respectively. Both lipopeptides can stimulate collagen proliferation, albeit to differing extents. The assay for C_16_–KTTKE shows statistically significant decreases in total collagen production (wt %) for concentrations above 0.003125 wt %, whereas C_16_–KTTKY shows an increase in collagen for the same concentrations. The values obtained for the total collagen production are in line with previous reports for C_16_–KTTKS using this technique.^35^

**Figure 7:**
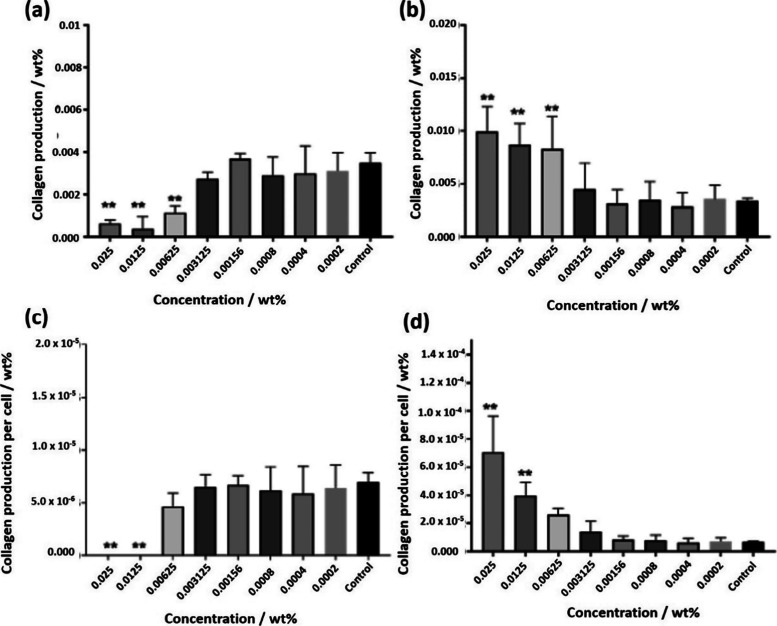
Total collagen deposited was quantified with the help of the picrosirius for (a) C_16_–KTTKE, (b) C_16_–KTTKY. The production of collagen per cell is shown in (c) and (d) for C_16_–KTTKE and C_16_–KTTKY, respectively. The statistical test used for data treatment was ANOVA, *n* = 3 with Bonferroni correction multiple comparisons, with ** *p* < 0.01.

Opposing effects were observed for the sequences, with a decrease in total collagen production at high concentrations for C_16_–KTTKE and an increase of almost 3-fold for C_16_–KTTKY. When we compare the ratio between collagen and the estimated number of viable cells by MTT, the differences are even more noticeable. In Figure 7c, a decrease in collagen per cell is observed above 0.00625 wt % due to the fact that there were no viable cells at the end of the incubation with C_16_–KTTKE. Figure 7d shows the opposite effect, with almost a 10-fold increase in the collagen per cell at higher concentrations. This effect became more noticeable due to cytotoxicity at increasing concentrations of lipopeptide, since the total collagen produced was similar between the concentrations of 0.025, 0.0125, and 0.00625 wt %, i.e., collagen production stimulation with HDFa cells saturates at a concentration of 0.00625 wt % of C_16_–KTTKY.

This result reflects concentration-dependent cytotoxicity that develops over the 72 h incubation rather than immediate cell death. MTT measures metabolic activity and not direct apoptosis or necrosis, unlike a Live/Dead assay. Consequently, even if fibroblasts produced more collagen early after exposure, the subsequent impact of higher compound concentrations on cell physiology and metabolism rendered the cells nonviable; however, the collagen deposited during incubation remained in the well and was quantifiable by Picrosirius red. A 72 h incubation (instead of 24 h) has been used to capture such longer-term effects on overall cell health, as observed in this study and previous studies.^35^

The assay shows that C_16_–KTTKY stimulates collagen synthesis in human dermal fibroblasts. This can be associated with the interaction of l-tyrosine residues, mainly in the O-sulfated form, with extracellular matrix proteins, such as dermatopontin and fibromodulin. These interactions can influence the self-assembly and, consequently, the enhancement of collagen fibril formation.^44,63,64^ This result indicates that this lipopeptide has the potential to increase skin firmness and to have an antiwrinkle effect.

*Staphylococcus epidermidis* is a Gram-positive commensal bacterium that plays crucial roles among the symbiotic microbiota, and the effect of the lipopeptides on this strain were examined. A study conducted by Zheng et al. demonstrated the importance of *S. epidermidis* in preserving the skin’s barrier function.^65^ A rapid *in vitro* growth of *S. epidermidis* microorganisms was observed under aerobic conditions with a peak at 24 h (Figure S2), decreasing thereafter.

The agar growth inhibition test shows the direct effect of the peptides on *S. epidermidis*, and the results were compared with the positive bacterial control (bacteria in culture medium). Generally, short ionic lipopeptides display antibacterial activity.^66–70^ Specifically for KTTKS sequences, Gomes *et al.* found a strong antibacterial activity for KTTKS lipopeptides with imidazole-functionalized ionic liquids installed at the N-terminus or on lysine side chains.^71^ However, the C_16_–KTTKE and C_16_–KTTKY lipopeptides did not inhibit bacterial growth either in the well or on film paper (Figure 8a–8d), on the contrary, an increase in bacterial cells was observed for all concentrations studied (all above the *cac*) (***p > 0.001) and the optical density (OD) was higher for the C_16_–KTTKY peptide than in C_16_–KTTKE (Figure 8e,8f), indicating that the lipopeptides can stimulate the growth of microorganisms and promote healthy microbiota.

**Figure 8:**
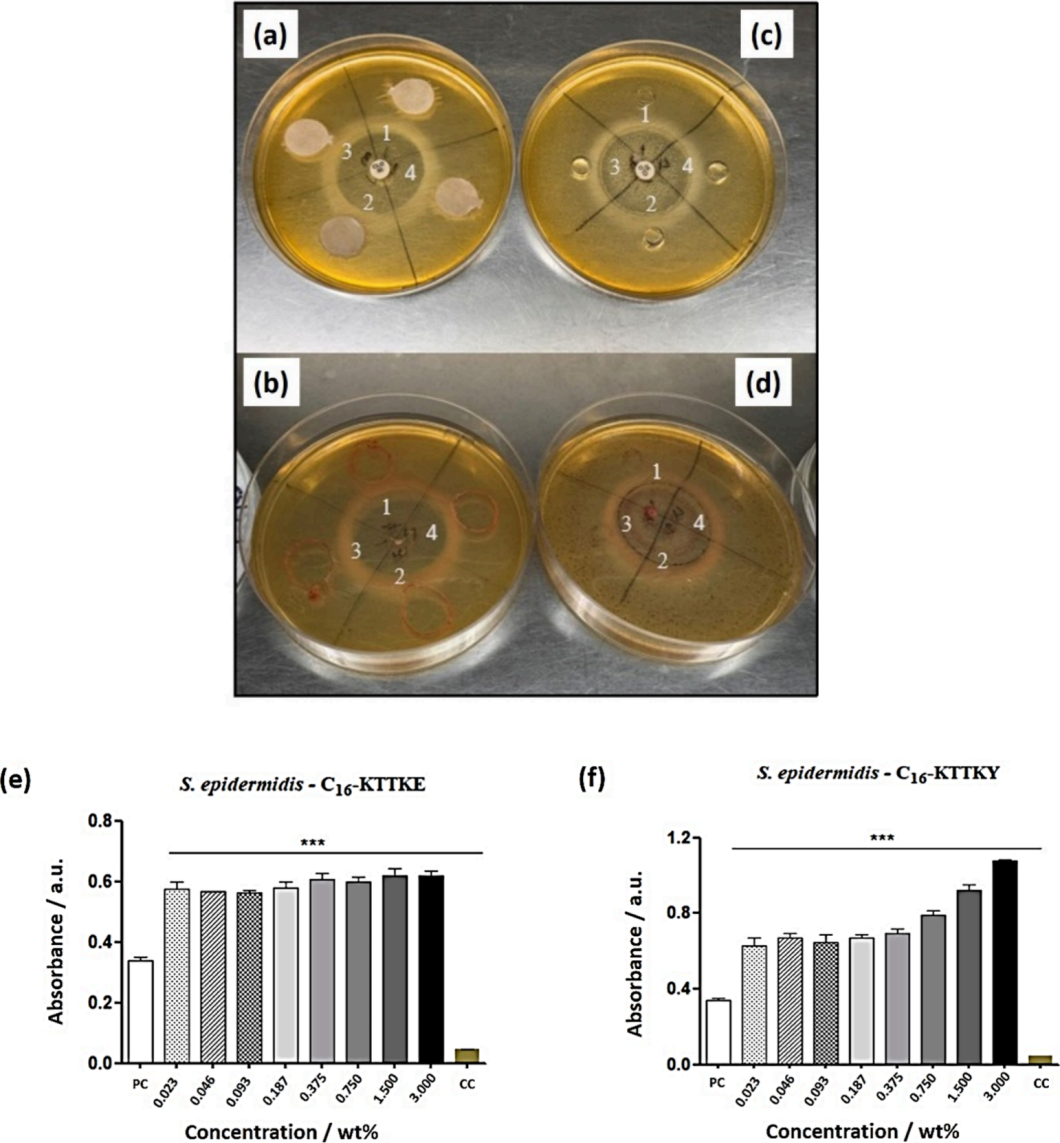
Inhibition of growth of *S. epidermidis*: (a) film paper before TTC staining (1 and 2: C_16_–KTTKE; 3 and 4: C_16_–KTTKY; (b) inhibition by film paper, after TTC staining (1 and 2: C_16_–KTTKE; 3 and 4: C_16_–KTTKY); (c) inhibition by well, before TTC staining (1 and 2: C_16_–KTTKE; 3 and 4: C_16_–KTTKY) and (d) after TTC staining (1 and 2: C_16_–KTTKE; 3 and 4: C_16_–KTTKY). Microdilution tests: (e) C_16_–KTTKE and (f) C_16_–KTTKY. PC: positive control (medium + microorganisms) and CC: cell control (medium). ANOVA test ***p < 0.001.

The different behavior of the lipopeptides can be associated with the distinct C-terminal residues in the two lipopeptides, which are substrates for nutrition and the formation of other compounds of interest. Literature reports indicate that l-amino acids favor the development of biofilm.^72–74^ We suggest that the l-glutamic acid residue in C_16_–KTTKE can facilitate the growth and survival of *S. epidermidis*, in the same way that the bacterium secretes poly-γ-glutamic acid (PGA), which protects it from environmental stresses and from the immune system, increasing bacterial persistence.^75^ However, for l-tyrosine present in C_16_–KTTKY, this correlation is not direct. It is suggested that this residue can increase *S. epidermidis* growth by serving as an essential nutrient source for protein synthesis, acting as a precursor for metabolic processes, leading to its growth and biofilm formation.

Byrd and colleagues demonstrated the role of this bacterium in atopic dermatitis (AD).^76^ The results suggest that AD in children was characterized predominantly by an increase in the level of *Staphylococcus aureus* for the most severe manifestations and *S. epidermidis* for the less severe manifestations. There is competition for space and resources between both microorganisms, and *S. epidermidis* can inhibit *S. aureus* by the production of small molecules that disrupt *S. aureus* virulence factors. A recent study showed that a strain of *S. epidermidis* inhibited the uptake of *S. aureus* by keratinocytes in AD.^77^ Furthermore, *S. epidermidis* produces a protease called Esp, which, when purified, inhibits biofilm formation and destroys preexisting *S. aureus* biofilms.^78^ Our results are of interest in the context of control of *S. epidermidis* as a way to preserve the skin barrier function.

The antioxidant activity of the lipopeptides was tested using the DCFH-DA dye, which allowed for the estimation of intracellular generation of reactive oxygen species. Resveratrol (250 μM) served as a positive control, and hydrogen peroxide (H_2_O_2_) as a negative control. The results can be seen in Figure 9. We observed a better antioxidant property for C_16_–KTTKE than C_16_–KTTKY, the former exhibiting similar characteristics to resveratrol at higher concentration (0.1293 wt %).

**Figure 9:**
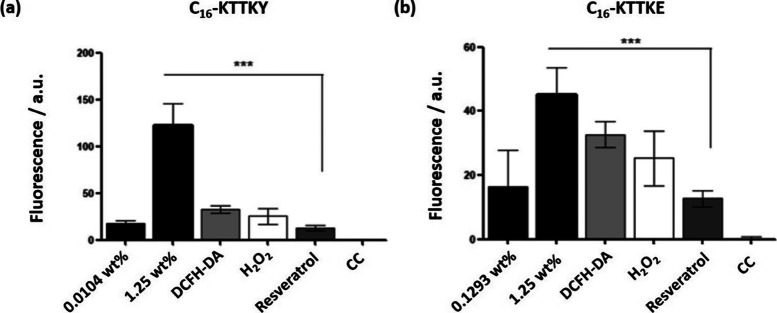
Antioxidant activity using two different concentrations of lipopeptides C_16_–KTTKY (a) and C_16_–KTTKE (b) lipopeptides. Fluorescence obtained with the ROS test in comparison to resveratrol 250 μM. Resveratrol was used as a positive control, H_2_O_2_ as a negative control, and CC as cell control. ANOVA test ***p < 0.001.

In antioxidant activity assays, 2′,7′-dichlorodihydrofluorescein (DCFH) was used as a redox-sensitive probe to estimate the intracellular or chemical oxidative activity. DCFH is a nonfluorescent compound that is readily oxidized by reactive oxygen species (ROS), particularly hydrogen peroxide–derived radicals in the presence of peroxidases or metal ions, forming the fluorescent product 2′,7′-dichlorofluorescein (DCF). Antioxidant compounds reduce the level of oxidation of DCFH by scavenging ROS or inhibiting radical-generating reactions, leading to decreased DCF fluorescence. Therefore, the assay indirectly reflects antioxidant capacity through the inhibition of DCF formation rather than direct measurement of specific ROS. When the scales are considered, the peptides exhibited different fluorescence intensities as apparent in Figure 9.^79^

Above 1 wt % for either lipopeptide, oxidant characteristics were noted, more closely resembling the behavior of H_2_O_2_. The results indicate antioxidant properties for both lipopeptides at lower concentrations. Some antioxidant compounds exhibit a nonlinear, concentration-dependent behavior. At higher concentrations, changes in the chemical properties of the molecules may occur, leading to reduced radical-scavenging efficiency or even the emergence of pro-oxidant activity rather than antioxidant effects. This phenomenon is associated with chemical hormesis, in which low doses of a substance exert beneficial effects, whereas higher doses result in diminished efficacy or opposite biological responses. Similar behavior was reported in the study conducted by Nowak et al. with polyphenolic compounds.^79^ Possible concentration-dependent autofluorescence may also be a factor, considering the data for C_16_–KTTKY.

## Discussion

We have investigated the aggregation and self-assembly of lipopeptides C_16_–KTTKY and C_16_–KTTKE, differing only in the last amino acid residue. Above the *cac*, both lipopeptides are characterized by a β-sheet secondary structure and a nanotape morphology. The lipopeptides unexpectedly show major differences in collagen stimulation and antioxidant properties: C_16_–KTTKE presented a lower collagen production above 0.00625 wt % peptide, however a better antioxidant characteristic (at 0.1293 wt %); while the opposite effect was observed for C_16_–KTTKY, which showed a significant increase in the collagen stimulation for the corresponding concentrations and a lower antioxidant characteristic (at 0.0104 wt %). Despite the significant reduction in viability of HDFa cells for both lipopeptides at high concentration, stimulation of bacterial cells, i.e., *S. epidermidis*, was noted at concentrations where cytotoxicity to eukaryotic HDFa cells was observed. We propose that C_16_–KTTKE can serve as a glutamic acid–based *S. epidermidis* coating and C_16_–KTTKY can act as a precursor for metabolic processes, leading to *S. epidermidis* growth and biofilm formation. These findings suggest that controlling these two parameters is essential for maintaining skin integrity.

Given these findings, it is reasonable to suggest that further studies with the lipopeptides C_16_–KTTKE and C_16_–KTTKY should be conducted to clarify their role in skin collagen regeneration and as an active ingredient to regulate commensal microbiota. These lipopeptides have potential in new cosmeceutical products that increase *S. epidermidis* production and, for C_16_–KTTKY, stimulation of collagen production to maintain and preserve the skin barrier function.

## Supplementary Material



## Notes

Additional cryo-TEM image and cross-sectional analysis, growth curve of *S. aureus*, table of SAXS fitting parameters (PDF)
