# Supplementary material for: Novel Palmitoyl Pentapeptides: Self-Assembly, Collagen Stimulation, Antioxidant Activity, and Influence of Skin Microbiota Health
Source: ACS Omega. 2026 Mar 20;11(13):20691–702. doi: 10.1021/acsomega.5c12526 (PMC13063098; doi:10.1021/acsomega.5c12526)
Supplement: Supplementary file 1 [file ao5c12526_si_001.pdf]

Supporting Information

Novel Palmitoyl Pentapeptides: Self-Assembly, Collagen Stimulation and Antioxidant Activity and Influence of Skin Microbiota Health

Juliane N. B. D. Pelin [a], Lucas R. de Mello [b], Raquel Allen Garcia Barbeto Siqueira [a,c], Matheus de Souza Alves [a], Valeria Castelletto [b], João Francisco Almeida [d], Jani Seitsonen [e], Patricia Santos Lopes[a], Vânia Leite-Silva [a], Newton Andreo-Filho [a], Ian W. Hamley [b],*

[a] Departamento de Ciências Farmacêuticas, Universidade Federal de São Paulo, 09913-030, Diadema, São Paulo, Brazil.

[b] Department of Chemistry, University of Reading, Reading RG6 6AD, United Kingdom.

[c] Programa de Pós-Graduação em Medicina Translacional, Departamento de Medicina, Escola Paulista de Medicina, Universidade Federal de São Paulo, São Paulo 04021-001, Brazil;

[d] Centro de Ciências Naturais e Humanas, Universidade Federal do ABC, 09210-580, Santo André, Brazil.

[e] Nanomicroscopy Center, Aalto University, Puumiehenkuja 2, FIN-02150 Espoo, Finland

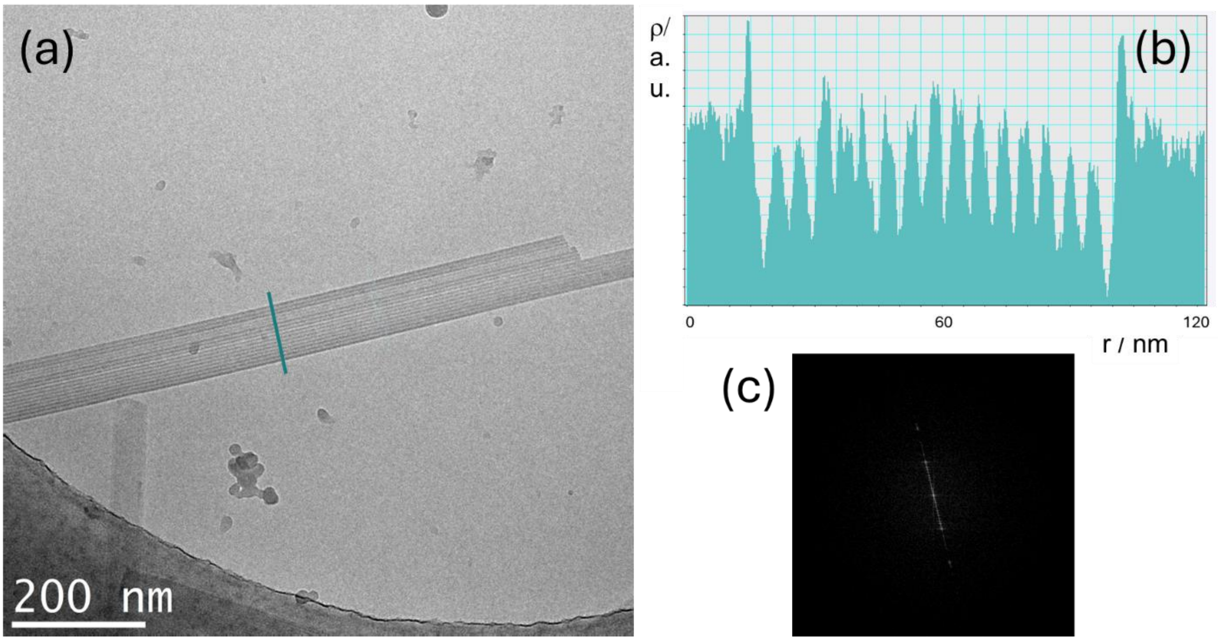

Figure S1. (a) Cryo-TEM images showing striped nanotapes for C_{16}-KTTKY (1 wt% solution) along with (b) cross-section density profile and (c) FFT of image.

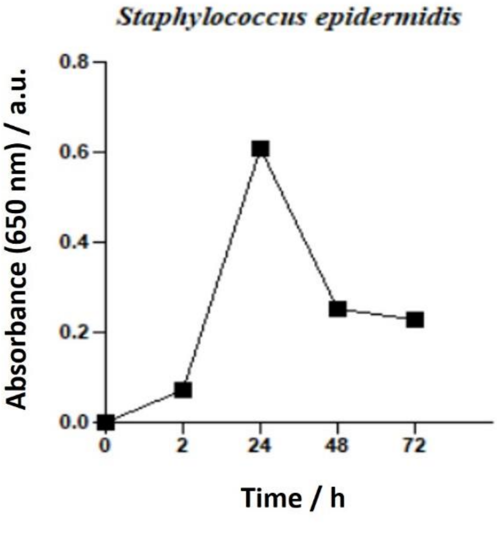

Figure S2. Growth curve of microorganism *Staphylococcus epidermidis* during 72 hours**.**

Table S1. Parameters extracted from the fitting of the SAXS data for a 1 wt% solution of C_{16}-KTTKE.[a]

	**C_{16}-KTTKE, 1 wt%**
$t \pm \Delta t$ [Å]	30.8 ± 3.2
r_H [cm^{-1}]	-2.10×10^{-6}
σ_H [Å]	7.4
r_C [cm^{-1}]	-5.44 ×10^{-7}
σ_C [Å]	73.8
D [Å] [b]	807
N	1.9
D [Å]	47.4
η	0.10
ν	0.28
C [cm^{-1}]	1.19×10^{-3}

[a] Data fitted using the software SASfit.[1-2]

[b] Fixed Parameter

Key: Gaussian bilayer: half layer thickness t (Gaussian polydispersity Δt), scattering contrast of outer (headgroup) layers r_H, and core (lipid chain) layer r_C, Gaussian widths σ_C and σ_H of core and headgroup layers respectively, D diameter (width) of layer system (when $D >> t$ as here, it acts as a scaling parameter for the form factor). **Lamellar structure factor (Caillé model[3]):** N number of layers, d layer spacing, η lamellar fluctuation parameter, ν diffuse scattering term. **Background:** constant background, C.

References

(1) Bressler, I.; Kohlbrecher, J.; Thünemann, A. F., SASfit: a tool for small-angle scattering data analysis using a library of analytical expressions. *J. Appl. Cryst.* **2015,** *48*, 1587–1598.

(2) Kohlbrecher, J.; Bressler, I., Updates in SASfit for fitting analytical expressions and numerical models to small-angle scattering patterns. *J. Appl. Cryst.* **2022,** *55*, 1677–1688.

(3) Caillé, A., X-Ray Scattering by Smectic-A Crystals. *Comptes Rendus Hebdomadaires des Seances de'l Academie des Sciences Serie B* **1972,** *274* (14), 891–893.
